# Paragangliome tympanique simulant un cholestéatome: à propos d’un cas

**DOI:** 10.11604/pamj.2017.28.169.13033

**Published:** 2017-10-20

**Authors:** Bouchra Rafiq, Ghizlane El Mghari

**Affiliations:** 1 Service d’Endocrinologie-Diabétologie, Maladies Métaboliques et Nutrition, Laboratoire PCIM, FMPM, Université Cadi Ayyad, CHU Mohamed VI, CHU Mohamed VI, Marrakech, Maroc

**Keywords:** Paragangliolme, cholestéatome, acouphène pulsatile, Paragangliolma, cholesteatoma, pulsatile tinnitus

## Abstract

Tympanic paragangliomas are common benign tumors of the middle ear, made up of neuroendocrine cells dispersed along the major vascular axes of the head, the neck and the vertebral column. The revealing symptoms are hearing loss and a pulsatile tinnitus. Otoscopy often shows pulsatile retrotympanic reddish mass. MRI and somatostatin analogue scintigraphy (OctreoScan) are the imaging modality of choice. OctreoScan allows physicians to detect other tumor locations. The dosage of urinary methoxyl derivatives is determined, complemented by the screening of other components of multiple endocrine neoplasias (MEN). Biopsy is contraindicated because of the risk of hemorrhage. Surgery is the treatment of choice. We report the case of F. A., a 75-year old patient, admitted with tympanic paraganglioma revealed by hearing loss in his right ear (occurred 4 years before) associated with pulsatile tinnitus concomitant with the heart beating in his ear. He underwnt otoscopy showing retrotympanic reddish pulsatile mass in his right ear (A). (B) MRI of the brain showed total involvement of the middle ear and of the mastoid cells, without identifiable tumoral process, with distinct hyperintensity on diffusion-weighted imaging and strong contrast-enhancement. MEN assessment was without abnormalities, including 24h methoxyl derivatives, parathyroid hormone and calcitonin. The diagnosis of paraganglioma was retained because of the strong contrast-enhancement. OctreoScan was indicated before surgery.

## Image en médecine

Les paragangliomes tympaniques sont des tumeurs bénignes fréquentes de l’oreille moyenne, faites de cellules neuroendocrines, dispersées le long des axes vasculaires de la tête, du cou et de la colonne vertébrale. Une hypoacousie et un acouphène pulsatile en sont souvent les symptômes révélateurs. L’otoscopie révèle souvent une masse rougeâtre pulsatile rétro-tympanique. L’IRM et la scintigraphie aux analogues de la somatostatine (octréo-scan) sont les examens radiologiques de choix. L’octréo-scan permet de rechercher les autres localisations. Le dosage des dérivés méthoxylés urinaires est nécessaire, complété par le dépistage des autres composantes des néoplasies endocriniennes multiples (NEM). La biopsie est contre-indiquée, vu le risque hémorragique. La chirurgie est le traitement de choix. Nous rapportons le cas du patient FA, 75 ans, admis pour paragangliome tympanique, révélé par une hypoacousie droite installée depuis 4 ans, associée à des acouphènes pulsatiles concomitantes aux battements cardiaques. Otoscopie: masse rétro-tympanique droite rougeâtre battante (A). (B) Comblement totale de l’oreille moyenne et des cellules mastoïdiennes, sans processus tumoral individualisable, avec hypersignal franc en diffusion, qui se rehausse de façon intense après injection de produit de contraste (PDC). Le bilan de NEM s’est révélé sans anomalies, notamment les dérivés méthoxylés de 24h, parathormone et calcitonine. Le diagnostic de paragangliome a été retenu, vu que le rehaussement intense après injection de PDC. Le patient est prévu pour octréo-scan avant la chirurgie.

**Figure 1:**
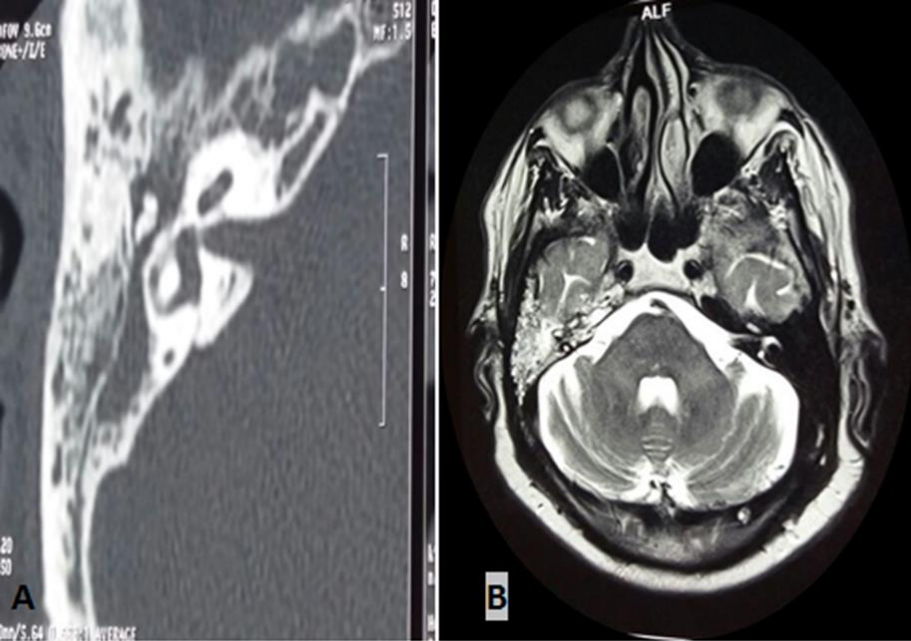
A) TDM du Rocher: otite moyenne droite avec comblement des cellules mastoïdiennes; B) IRM cérébrale

